# Rhythmic Spatial Self-Organization of Bacterial Colonies

**DOI:** 10.1128/mbio.01703-22

**Published:** 2022-08-08

**Authors:** Francesca Sartor, Ákos T. Kovács

**Affiliations:** a Institute of Medical Psychology, Faculty of Medicine, LMU Munich, Munich, Germany; b Bacterial Interactions and Evolution group, DTU Bioengineering, Technical University of Denmark, Kgs Lyngby, Denmark

**Keywords:** bacteria, circadian rhythm, colony, pattern, spatial organisation

## Abstract

Bacteria display a remarkable capacity to organize themselves in space and time within biofilms. Traditionally, the spatial organization of biofilms has been dissected vertically; however, biofilms can exhibit complex, temporally structured, two-dimensional radial patterns while spreading on a surface. Kahl and colleagues report a ring pattern that indicates the alternating redox metabolism of P. aeruginosa biofilms under light/dark cycles. Does the presence of a rhythmic, daily phenotype imply a circadian rhythm? Here, we highlight several examples of rhythmic patterns reported in the literature for surface-colonizing multicellular assemblies and discuss the conceptual requirements for proving the presence of a prokaryotic circadian clock behind pattern formation.

## COMMENTARY

**FIG 1:**
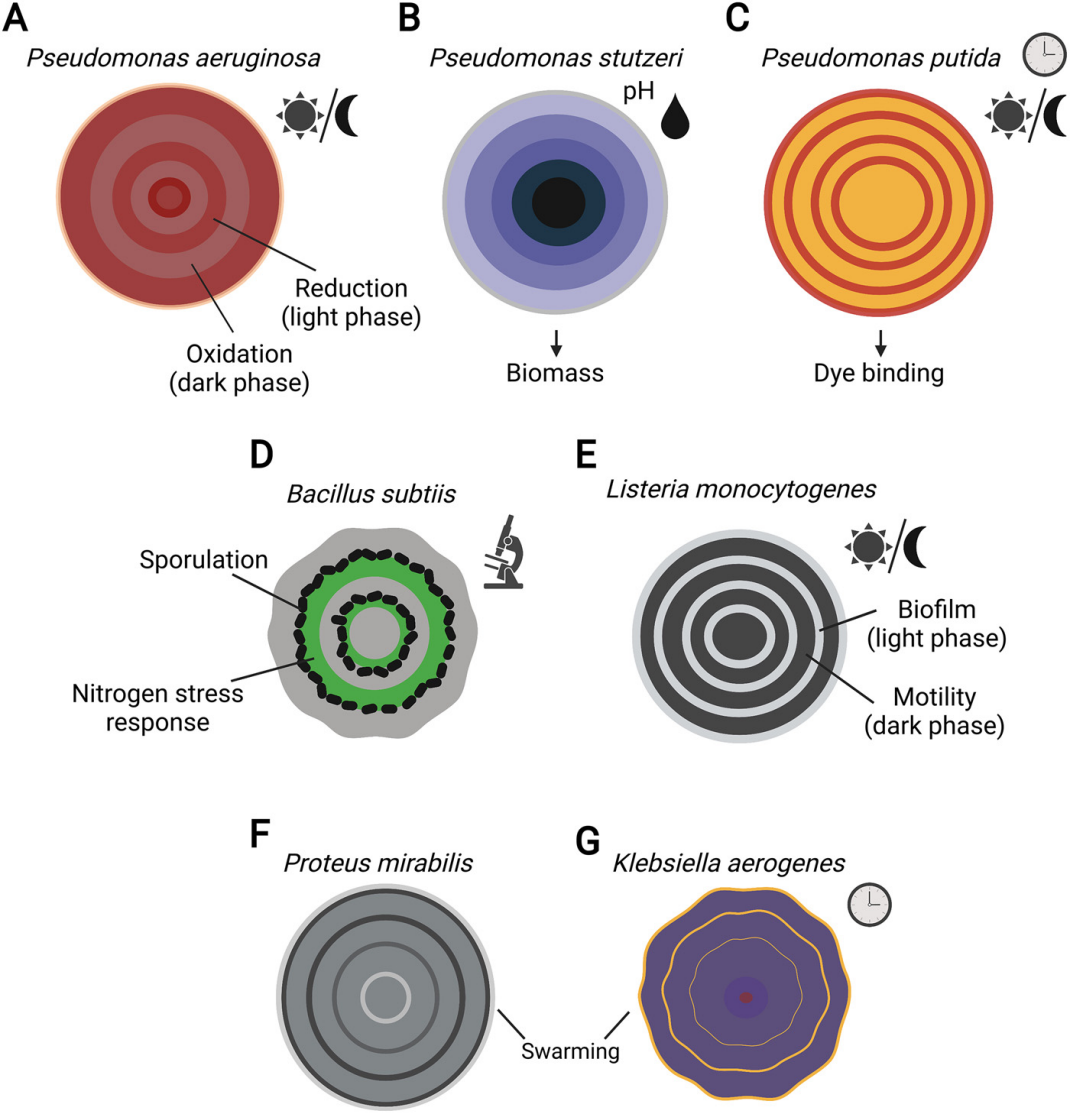
Examples of radial colony patterns that have been previously described for various microbes are highlighted. Different mechanisms can underlie the patterning, including (A) redox metabolism in P. aeruginosa (7); (B) expansion of the biomass of two metabolically interacting Pseudomonas stutzeri strains in response to cycling oxic/anoxic conditions (15); (C) dye-binding in P. putida (9); (D) sporulation and the nitrogen stress response in B. subtilis microcolonies (6); (E) developmental switch between motility and biofilm formation in Listeria monocytogenes (16); and (F and G) swarming behavior of Proteus mirabilis (17) and K. aerogenes (10). In P. putida and K. aerogenes, the cyclic changes described have been linked to the presence of a circadian clock. The figure was created on BioRender.com.

Microbes display various spatial organizations, even when cultivated under defined laboratory conditions. Such spatial heterogeneity develops in microbial populations or multispecies communities when growing as multicellular assemblies, either from bottom to top (e.g., stratification in 3D structures of biofilms and microbial mats) or from the centrum outward (e.g., expanding colonies on the surface of an agar-solidified medium). The spatial heterogeneity of biofilms is driven by resource gradients that lead to metabolic diversity and, vice versa, the gradients are also influenced by the metabolism (1). For example, oxygen availability in the biofilms drives the differentiation of Pseudomonas aeruginosa subpopulations that use different metabolic pathways connected by phenazines, which are redox balancing mediator molecules (2). Also, the phenotypic differentiation of bacterial populations becomes spatially structured in biofilm colonies, e.g., the spatial distribution of motile, matrix-producing, and spore-forming cell types of Bacillus subtilis (3) and curli-producing and flagellated cells of Escherichia coli (4). In addition to biofilm stratification, bacterial populations may display diverse radial patterns when expanding on an agar-solidified surface, e.g., during various motility types (5) or during complex colony formation (see examples in Fig. 1). Intriguingly, the spatial patterns of these expanding colonies display remarkable periodic cycles when observed from above. Such spatial self-organization could either occur in a fluctuating environment or develop under constant conditions (e.g., alternating sporulation and nitrogen stress response in Bacillus subtilis colony sections [6]). Kahl et al. (7) demonstrate that light-dark and temperature cycling modulate the redox metabolism in P. aeruginosa PA14 biofilms, displaying cyclic phenotypic patterns.

Are these approximately 24 h cycling phenotypes evidence of circadian rhythms? The answer is not trivial (8). Whereas they might indeed hint at the presence of a daily temporal program, one must distinguish “driven oscillations” from “circadian oscillations”.

Microbial gene expression or phenotypic traits might fluctuate as a response to periodic, environmental cycles without being under circadian control. Importantly, when driven, the oscillations will not persist in the absence of external stimuli. On the contrary, circadian clocks are endogenous timing systems. They can predict and anticipate periodic environmental changes and will continue to exhibit approximately 24 h self-sustained oscillations (called “free-running oscillations”), even in the absence of any external time cue. We can speculate that, as seen in higher forms of life, the adaptation of physiology and metabolism to cope with cycling, daily stress factors and the anticipation of nutrient availability could represent major forces driving the evolution of microbial circadian clocks and could thereby be contributing to the fitness of the microorganisms. For instance, stress factors for bacteria might be represented by fluctuations in temperature, pH, oxygen availability, or humidity in the soil. Also, light can be sensed by bacteria either directly, when bacteria are found near the soil surface or colonizing the above ground structures of plants, or indirectly, through the effect of light on the host.

The presence of free-running oscillations was first tested in a nonphotosynthetic bacterium, Pseudomonas putida, about 10 years ago (9). Cultures were grown under 24 h light-dark (LD) cycles on plates with dyes binding to proteins or extracellular polymeric substances. The growth pattern was synchronized with the LD cycles and persisted when the plates were subsequently kept in darkness (Fig. 1). This was surprising at the time, as nonphotosynthetic bacteria were generally considered to lack circadian machinery. Whether P. aeruginosa exhibits self-sustained oscillations in constant darkness following growth under LD cycles has not been tested, as an investigation of the circadian clock was not within the scope of the study by Kahl and colleagues (7). Nonetheless, one might wonder if this would be the case, as it could provide evidence for endogenous rhythmicity. The growth of P. aeruginosa colonies was only assayed when placed in constant light or dark conditions from the beginning of the experiment. Using these settings, no rings were formed. However, this should not be interpreted as proof against circadian rhythms. It is possible that each cell in the biofilm may possess a clock, but if the single cells’ clocks are out of phase, no rhythms will be discernible at the population level. The lack of a rhythmic output might therefore reflect asynchrony in the population rather than arrhythmicity. In chronobiological protocols, rhythms in constant conditions are typically examined by following the synchronization of samples with environmental cycles or by the addition of a synchronizing agent. For instance, as discussed above, the presence of a free-running rhythm is observed in the ring formation of P. putida following growth under LD cycles ([9] and Fig. 1). When cultures were placed under constant light or darkness from the beginning of the experiment, the ability to form rings was lost in most cultures. Among synchronizing agents, melatonin has been shown to contribute to the synchronization of circadian rhythms in animals. Interestingly, the addition of melatonin is required for the circadian swarming behavior of Klebsiella aerogenes, and it also synchronizes circadian reporter gene expression (10) (Fig. 1), suggesting that melatonin may also act as a synchronizer for the circadian rhythms of enteric bacteria.

Circadian rhythms should not be studied only under constant conditions. Circadian clocks of living organisms have evolved and function in cycling environments, and their true natures should also be dissected under these conditions. The characterization of a circadian system should, therefore, always consider entrainment properties. The process of entrainment goes beyond passively synchronizing with external conditions. Rather, it is a dynamic process in which the endogenous oscillator actively tries to find a stable phase relationship with the external environmental cycle.

Chronobiological protocols have been developed to allow us to understand whether a system exhibiting daily oscillations is: (i) simply responding to environmental changes (driven system) or (ii) entrained by environmental time cues (called the “zeitgeber”) (circadian system). So far, chronobiological principles to explore entrainment have been applied only in two nonphotosynthetic prokaryotic species: K. aerogenes (11) and B. subtilis (12). In both species, entrainment properties were elegantly explored using classical chronobiological protocols, such as systematic changes in phase angles under different, non-24 h entraining cycles (T-cycles). Another classical protocol by which to investigate entrainment is frequency demultiplication. When circadian systems are placed in entrainment cycles having a duration close to half of the endogenous period, they can entrain to every second cycle; that is, they demultiply. Interestingly, such a protocol was applied to P. aeruginosa by Kahl et al. (7), although with a different aim. From a visual examination of the biomass traces under 12 h LD cycles (6 h dark/6 h light), no obvious sign of frequency demultiplication was apparent. However, circadian components may emerge under detailed analyses and alternative methods for data representation.

In addition to entrainment and an approximately 24 h free-running period, circadian clocks are defined by the property of temperature compensation, meaning that the free-running period remains relatively constant within a broad range of temperatures. Among nonphotosynthetic bacteria, this has been demonstrated in K. aerogenes (10) and B. subtilis (12). Kahl and colleagues showed that Pseudomonas can form rings under temperature cycles ranging over a 10°C range, but it remains to be investigated whether the phenotype persists in free-running conditions and, if so, with what periodicity.

In brief, the observation of daily changes in P. aeruginosa biofilm colony patterning is remarkable. Are we observing a primordial redox oscillator (13)? As tempting as it might be, the reader should not automatically ascribe such rhythmic behavior to the presence of a circadian clock. A systematic analysis of circadian properties will be necessary to conclude such. Additionally, future experiments will be required to dissect rhythmic changes in the stratified structures of microbial assemblies and the role of the circadian clocks of nonphotosynthetic bacteria in driving gene expressions and metabolic functions. P. aeruginosa can integrate quorum sensing and light sensing to control virulence and biofilm formation (14). An understanding of whether these collective behaviors are under temporal, circadian regulation would have significant health and ecological implications.
